# Atomizing Apparatus

**Published:** 1868-09-15

**Authors:** 


### *Atomizing Apparatus.*

Codman & Shurtleff have furnished for experiment a specimen of their improved atomizing apparatus. It is fitted either for the purposes of local medication or refrigeration. A glass protecting funnel is added, which can be removed at pleasure, making the whole exceedingly compact and convenient. For sale by Bliss and Sharp, 144 Lake Street.

